# The Diurnal Variation in Mitochondrial Gene in Human Type 2 Diabetic Mesenchymal Stem Cell Grafts

**DOI:** 10.3390/ijms26020719

**Published:** 2025-01-16

**Authors:** Michiko Horiguchi, Kenichi Yoshihara, Yoichi Mizukami, Kenji Watanabe, Yuya Tsurudome, Kentaro Ushijima

**Affiliations:** 1 Division of Pharmaceutics, Faculty of Pharmaceutical Sciences, Sanyo-Onoda City University, Yamaguchi 756-0884, Japan; 2 Institute of Gene Research, Yamaguchi University Science Research Center, Yamaguchi 755-8505, Japan

**Keywords:** mitochondria, diurnal variation, mesenchymal stem cells (MSC), type 2 diabetes mellitus, mitophagy, NF-kB

## Abstract

The application of regenerative therapy through stem cell transplantation has emerged as a promising avenue for the treatment of diabetes mellitus (DM). Transplanted tissue homeostasis is affected by disturbances in the clock genes of stem cells. The aim of this study is to investigate the diurnal variation in mitochondrial genes and function after transplantation of adipose-derived mesenchymal stem cells (T2DM-ADSCs) from type 2 diabetic patients into immunodeficient mice. Diurnal variation in mitochondrial genes was assessed by next-generation sequencing. As a result, the diurnal variation in mitochondrial genes showing troughs at ZT10 and ZT22 was observed in the group transplanted with adipose-derived mesenchymal stem cells derived from healthy individuals (N-ADSC). On the other hand, in the group transplanted with T2DM-ADSCs, diurnal variation indicative of troughs was observed at ZT18, with a large phase and amplitude deviation between the two groups. To evaluate the diurnal variation in mitochondrial function, we quantified mitochondrial DNA copy number using the Human mtDNA Monitoring Primer Set, measured mitochondrial membrane potential using JC-1, and evaluated mitophagy staining. The results showed a diurnal variation in mitochondrial DNA copy number, mitophagy, mitochondrial membrane potential, and NF-kB signaling in the N-ADSC transplant group. In contrast, no diurnal variation was observed in T2DM-ADSC transplants. The diurnal variation in mitochondrial function revealed in this study may be a new marker for the efficiency of T2DM-ADSC transplantation.

## 1. Introduction

Mitochondria are essential cell organelles that regulate cell metabolism and serve as a source of cellular energy [1]. This includes the synthesis of ATP and fatty acids through oxidative phosphorylation. Mitochondria are produced by α-proteobacteria [2] and contain mitochondrial DNA (mtDNA), a circular genome that has been reduced by genetic transfer to the nucleus during evolution [3]. Mitochondria are dynamic cell organelles that fuse and divide, and the process of eliminating dysfunctional mitochondria through mitochondrial fusion, fission, and selective mitochondrial autophagy (mitophagy), collectively known as mitochondrial dynamics, is important in managing their quality and function [4].

Many metabolic genes show rhythms in their expression patterns and are regulated by the circadian clock system [5]. Circadian rhythms are regulated by clock genes and proteins such as period circadian regulator (PER), clock (CLOCK), cryptochrome (CRY), and aryl hydrocarbon receptor nuclear translocation factor-like (ARNTL or BMAL1), and these genes are essential for homeostasis [6]. Clock genes are present in most cells in the human body, and the central clock that controls these genes is located in the suprachiasmatic nucleus (SCN) [7]. Previous studies have analyzed clock gene function using models of diabetes and obesity [8,9]. Several studies have reported that long-term disruption of the phase and amplitude of clock genes in stem cells impairs tissue homeostasis [10,11,12]. Previous studies have shown that mitochondria involved in metabolism are no exception and are influenced by diurnal variations, optimizing mitochondrial function to match energy supply and demand [1]. Furthermore, mitochondrial dynamics and biogenesis are transcriptional targets of Bmal1 in the mouse liver, which has been shown to exhibit metabolic rhythms synchronized with diurnal bioenergetic demand [5].

Because mitochondrial function regulated by diurnal variations can influence physiological processes, it has also been shown to be associated with pathological conditions such as metabolic diseases, such as type 2 diabetes mellitus [13]. Diabetes mellitus (DM) is a chronic disease that is characterized by long-term abnormalities in glucose metabolism. This is due to a decrease in the secretion of insulin and an increase in insulin resistance [14,15]. The standard treatment for diabetes comprises lifestyle modifications and insulin replacement therapy [16]. In recent years, regenerative therapy involving stem cell transplantation has emerged as an effective treatment option in this context [17,18,19]. A variety of stem cells have been used in regenerative therapies for diabetes. For example, mesenchymal stem cells (MSCs), which are in stable supply, are transplanted to promote the secretion of growth factors and tissue regenerative factors. Adipose-derived mesenchymal stem cells (ADSCs) are characterized by their higher immunosuppressive potential compared to bone marrow-derived cells. Therefore, ADSCs are attracting attention as an excellent cellular material for regenerative therapy of patients with rapidly progressing severe diabetes mellitus.

A variety of stem cells is utilized in regenerative therapies for the treatment of diabetes [20]. For example, efforts have been made to transplant mesenchymal stem cells (MSCs), which are in stable supply, with the objective of promoting the secretion of growth and tissue regeneration factors [21]. MSCs can be obtained from a variety of tissues [22,23]. Adipose-derived mesenchymal stem cells (ADSCs) are characterized by a higher capacity to suppress the immune system than those derived from bone marrow (bone marrow-derived mesenchymal stem cells [BMSCs]) [24,25,26]. Nevertheless, it remains uncertain whether autologous ADSCs from patients with Type 2 diabetes can achieve a similar engraftment rate as ADSCs from healthy individuals.

We have previously reported on the evaluation of the ADSCs derived from a healthy individual (N-ADSC), ADSCs derived from a patient with type 1 diabetes mellitus (T1DM-ADSC), and ADSCs derived from a patient with type 2 diabetes mellitus (T2DM-ADSC) [27,28,29,30,31]. The results indicated hypertrophy of the nuclei of T2DM-ADSCs and degenerations of the mitochondrial cristae structures [27]. The expression of Emerin, a protein that constitutes the nuclear membrane, was observed to be low in T2DM-ADSCs, and a reduction in the activity of mitochondrial enzymes was also noted [27]. While these findings unambiguously demonstrate that the mitochondria of T2DM-ADSCs are in a state of degeneration, it is yet to be determined whether mitochondrial genes and functions undergo changes following transplantation. The objective of this study is to ascertain the diurnal variation in mitochondrial genes and function following the transplantation of stem cells derived from patients with T2DM. We have previously shown that diurnal variations in clock genes are maintained after ADSC transplantation [28]. However, the rhythms of two mitochondrial genes, MTATP8P1 and NDUFA7_2, were found to be significantly altered during the transplantation of ADSCs derived from type 2 diabetic patients [28]. Therefore, in this study, we further elucidated the 24-h diurnal variation in mitochondrial genes during stem cell transplantation.

## 2. Results

### 2.1. Between N-ADSC and T2DM-ADSCs Transplants, Daily Fluctuations in Mitochondrial Genes Showed Variations in Phase

**Figure 1:**
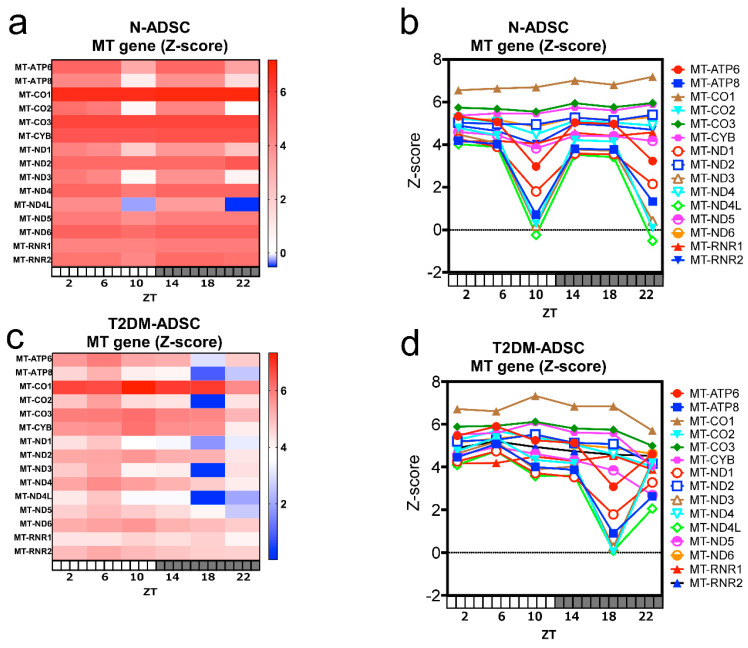
Daily fluctuations in mitochondrial gene expressions. (**a**,**b**) Daily fluctuations in mitochondrial genes in adipose-derived mesenchymal stem cells derived from healthy individuals’ transplants. (**c**,**d**) Daily fluctuations in mitochondrial genes in adipose-derived mesenchymal stem cells derived from a patient with type 2 diabetes mellitus transplants. (**a**,**c**) Heat map of mitochondrial gene expression. (**b**,**d**) The 24-h sequential monitoring graph of mitochondrial gene expression. Abbreviations in figure: N-ADSC, adipose-derived mesenchymal stem cells derived from a healthy individual; T2DM-ADSC, adipose-derived mesenchymal stem cells derived from a patient with type 2 diabetes mellitus; ZT, Zeitgeber time; MT, mitochondrially encoded.

Fifteen highly expressed mitochondrial genes were identified, namely, mitochondrially encoded ATP synthase membrane subunit 6 (*MT-ATP6*), *MT-ATP8*, mitochondrially encoded cytochrome c oxidase I (*MT-CO1*), *MT-CO2*, *MT-CO3*, mitochondrially encoded cytochrome b (*MT-CYB*), *MT-ND1*, *MT-ND2*, *MT-ND3*, *MT-ND4*, *MT-ND4L*, *MT-ND5*, *MT-ND6*, mitochondrially encoded 12S rRNA (*MT-RNR1*), and mitochondrially encoded 16S rRNA (*MT-RNR2*) (Figure 1). In the N-ADSC transplant, the mitochondrial genes *MT-ATP6*, *MT-ATP8*, *MT-CO2*, *MT-ND1*, *MT-ND3*, and *MT-ND4L* showed daily fluctuations, reaching their lowest levels at ZT10 and ZT22 (Figure 1a,b). Moreover, daily fluctuations in the mitochondrial genes *MT-ATP6*, *MT-ATP8*, *MT-CO2*, *MT-ND1*, *MT-ND3*, *MT-ND4L*, and *MT-ND5* differed in phase between the T2DM-ADSC and N-ADSC transplants, reaching their lowest levels at ZT18. These results revealed that daily fluctuations in the mitochondrial genes differed in the phase between the N-ADSC and T2DM-ADSC transplants (Figure 1c,d).

### 2.2. Daily Fluctuations in Mitochondrial Function Differed in Phase and Amplitude Between the N-ADSC and T2DM-ADSC Transplants

**Figure 2:**
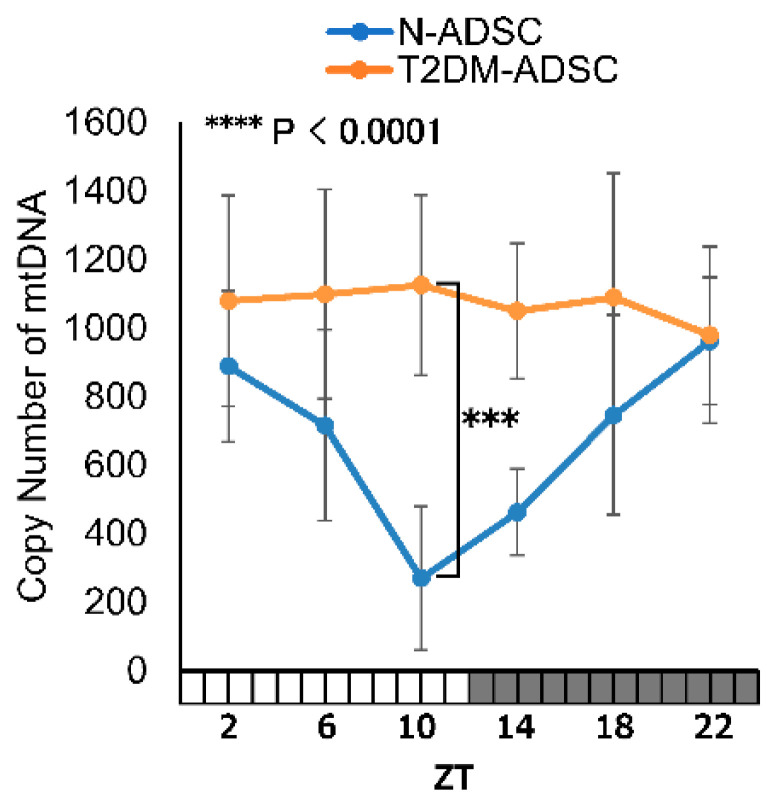
Daily fluctuations in the number of mitochondrial DNA copies. The number of mitochondrial DNA copies is represented by the mean of 2^ΔCt^ from the difference in the cycle threshold values of ND1/SLCO2B1 and ND5/SERPINA1. Light periods are indicated by the white column on the horizontal axis, and dark periods by the black column. **** *p*-values < 0.0001. *** *p*-values < 0.005. Data are shown as the mean ± SD; n = 6 for each sampling point, normally distributed; two-way ANOVA with Tukey–Kramer post hoc test.

We explored the characteristics through which T2DM affected the daily fluctuations in mitochondrial function. We decided to analyze the daily fluctuations in the number of mtDNA copies. The N-ADSC group exhibited daily fluctuations in the number of mtDNA copies, reaching the lowest number at ZT10. In contrast, daily fluctuations were not observed in the T2DM-ADSC transplant (Figure 2).

**Figure 3:**
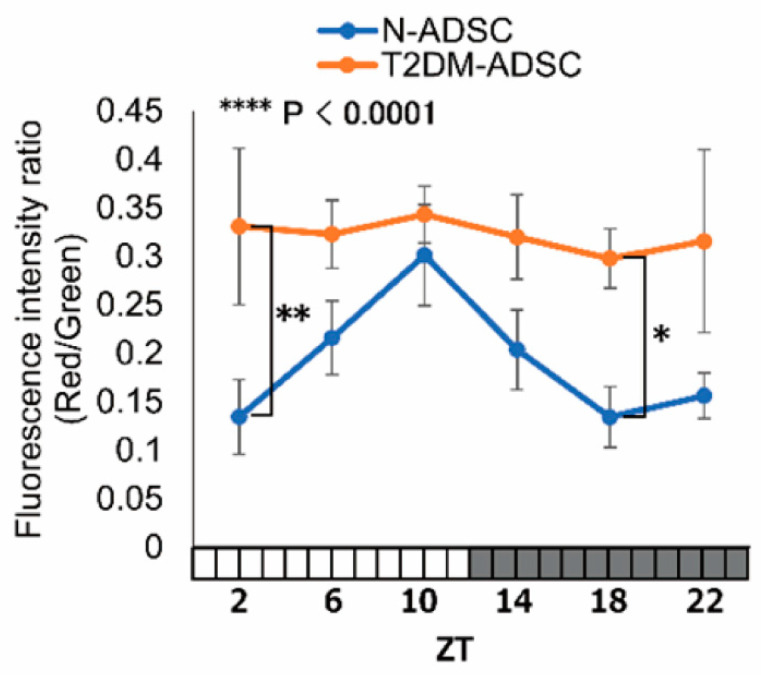
Daily fluctuations in mitochondrial membrane potential. The difference in mitochondrial membrane potential is represented by the ratio of red fluorescence intensity to green fluorescence intensity. * *p*-values < 0.05. ** *p*-values < 0.01. **** *p*-values < 0.0001. Data are shown as the mean ± SD, n = 6 for each sampling point, normally distributed; two-way ANOVA with Tukey–Kramer post hoc test.

In this study, a low molecular fluorescent dye JC-1 was used to observe the mitochondrial membrane potential. Differences in mitochondrial membrane potential prompt the accumulation of JC-1 in mitochondria. In addition, it alters the fluorescence properties from green fluorescence (wavelength: 530 nm) to red fluorescence (wavelength: 590 nm) due to the accumulation of the dye. Thereafter, the ratio of red fluorescence intensity to green fluorescence intensity was evaluated. The fluorescence intensity ratio of JC-1 mitochondrial accumulation, which reflects mitochondrial membrane potential difference, showed daily fluctuations in the N-ADSC transplant, peaking at ZT10. However, the T2DM-ADSC transplant did not exhibit daily fluctuations (Figure 3).

**Figure 4:**
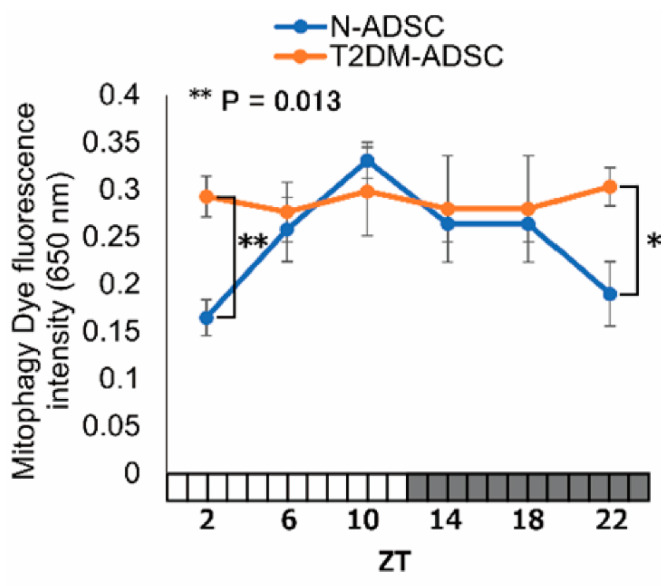
Daily fluctuations in mitophagy. Daily fluctuations in mitophagy, as displayed by the fluorescence intensity of the Mtphagy Dye that visualizes mitophagy. * *p*-values < 0.05. ** *p*-values < 0.01. Data are shown as the mean ± SD; n = 6 for each sampling point, normally distributed; two-way ANOVA with Tukey–Kramer post hoc test.

Mitophagy is a system that selectively eliminates mitochondria that are degraded due to oxidative stress and DNA damage. In mitophagy, degraded mitochondria are isolated by autophagosomes and fused with lysosomes, where they are finally digested. In this study, we used the fluorescence intensity of mitophagy dye (which visualizes mitophagy) to evaluate daily fluctuations in mitophagy. In the N-ADSC transplant, the fluorescence intensity of the mitophagy-visualizing dye showed daily fluctuations, peaking at ZT10. In contrast, no daily fluctuations were observed in the T2DM-ADSC transplant (Figure 4).

**Figure 5:**
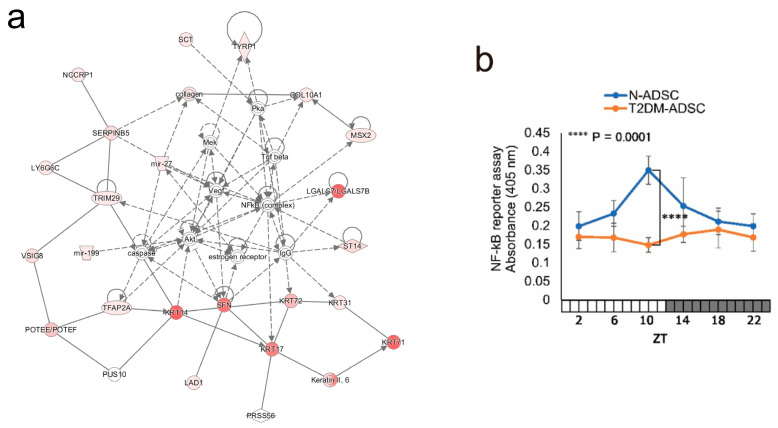
Daily fluctuations in nuclear factor-kB (NF-kB) signaling. (**a**) Schematic of the signaling pathway by next-generation sequencing. (**b**) Daily fluctuations in the NF-kB reporter activity. The NF-kB reporter activity is represented by absorbance levels measured using the NF-kB reporter assay. **** *p*-values < 0.0001. Data are shown as the mean ± SD; n = 6 for each sampling point, normally distributed; two-way ANOVA with Tukey–Kramer post hoc test.

Network analysis of Diseases and Functions by QIAGEN Ingenuity Pathway Analysis (IPA) was performed using diurnal variation genes at six time points. As a result, the top three networks extracted were Tissue Development, Molecular Transport, and Development and Function, and the NF-kB signal was extracted as a common factor (Figure 5a). It has been reported that the NF-kB pathway controls the balance of metabolic responses by regulating mitochondrial respiration. Therefore, we analyzed the diurnal variation in the NF-kB signal and found its homology with the circadian rhythm of mitochondrial function. NF-kB was identified as a factor that plays a central role in the extracted signaling pathway (Figure 5a). When daily fluctuations were evaluated using the NF-kB reporter assay, the results showed daily fluctuations in NF-kB reporter activity in the N-ADSC transplant, peaking at ZT10. Nevertheless, no daily fluctuations were observed in the T2DM-ADSC transplant (Figure 5b).

## 3. Discussion

This study investigated the 24-h diurnal variation in mitochondrial genes and functions after stem cell transplantation. The results revealed that mitochondrial genes and functions showed daily fluctuations only in the N-ADSC group; those were lost in the T2DM-ADSC group, revealing marked differences in phase and amplitude between the two types of transplants.

The diurnal variation in mitochondrial genes in the N-ADSC group showed twice daily variation with two troughs at ZT10 and ZT22. In contrast, the T2DM-ADSC group showed a single rhythm with a trough at ZT18. There have been no previous reports on the differences in this cycle. In this study, we found that the peak of NF-kB activity overlaps at ZT10, where the cycle change is most pronounced. The diurnal variation in mitochondrial function is large in N-ADSCs at ZT10. Mitochondrial DNA copy number showed a trough at ZT10, while mitochondrial membrane potential, mitophagy, and NF-kB peaked at ZT10. This diurnal variation was similar to that of the clock genes Cry1 and Bmal1 [5,6,7,8,9,10,11,12], suggesting that they were regulated by clock genes. On the other hand, the diurnal variation in mitochondrial function was absent in T2DM-ADSCs, indicating that diabetes induced diurnal variation in mitochondrial function.

The present findings may contribute to clarifying the daily fluctuations in clock genes and mitochondrial genes following stem cell transplantation. This study revealed that the diurnal variations in mitochondrial genes and functions were independent of those observed in clock genes. Moreover, T2DM-ADSC exhibited diurnal variations in mitochondrial genes and functions that were different from those of N-ADSC. These findings suggest that diabetes mellitus may be related to the diurnal variations in mitochondria in stem cells. In future studies, we intend to clarify the molecular regulatory mechanism underlying the diurnal variations in mitochondrial genes and functions in T2DM-ADSC. 

There are several limitations in this study. First, this study is a xenotransplantation model in which human ADSCs are transplanted into immunodeficient mice, so the effects of species differences cannot be avoided. There are some differences between humans and mice in genes, proteins, immune systems, etc. Therefore, to overcome this limitation, clinical trials need to be planned in the future. Secondly, because stem cell grafts are used for evaluation, it is technically difficult to clarify the molecular mechanism of rhythm control, and we have no choice but to infer the molecular mechanism through function analysis. Although this study identifies the molecular mechanism of NF-kB (Figure 5a), it is essential to develop a method to evaluate upstream factors and transcriptional regulators. 

We are examining methods such as primary culture of cells from stem cell grafts, but we are not able to secure sufficient cells at present, so we continue to work on developing methods. At present, we are using trypsin EDTA buffer containing STEMxyme 2-Collagenase (Worthington) and centrifugation at 1000× *g* for 20 min. We will continue to optimize the buffer and experimental conditions for more detailed studies. 

In summary, T2DM-ADSC exhibited diurnal variations in mitochondrial genes and functions that were different from those of N-ADSC. These findings suggest that diabetes mellitus may be related to the diurnal variations in mitochondria in stem cells. The diurnal variations in mitochondrial function revealed in this study may provide a new measure of the efficiency of T2DM-ADSC transplantation.

## 4. Methods

### 4.1. Materials

The materials and instruments used in this study are listed in Supplementary Table S1.

### 4.2. ADSC Transplantation Model

The ADSC transplantation model used in this study was established in our preceding studies [30,31]. ADSCs were cultured to form spheroids by adding a spheroid culture matrix (356255, Corning) one week prior to transplantation. Stem cell spheroids were mixed with transplantation matrix (354234, Corning) in a 1:1 ratio; ADSC spheroid solutions were filled into syringes on ice and transplanted into the back subcutaneous tissue of immunodeficient mice (BALB/cAJcl-*nu/nu*). Three weeks after transplantation, the skin grafts were removed and used for this study. Five-week-old female BALB/cAJCL-*nu/nu* mice were purchased from Clare Japan Inc. (Tokyo, Japan) and kept under normal rearing conditions with a 12 h light/12 h dark cycle. To analyze diurnal variation, the time of light onset (start of light period) was set to ZT0 (meaning 0 h in Zeitgeber time). Stem cell transplants were collected at 4-h intervals starting at ZT2.

This study was conducted according to the guidelines for the Proper Conduct of Animal Experiments and Related Activities in Academic Research Institutions and approved by the Institutional Review Board of Sanyo-Onoda City University (protocol code 2020-8-A, and date of approval 12 March 2020). Also, this study was conducted according to the ARRIVE guidelines.

This study used ADSCs that were ethically approved by Lonza. The ADSCs consent form was obtained at Lonza, where the cells were purchased.

N-ADSCs established by primary culture from adipose tissue of a healthy 23-year-old woman without underlying disease were used in this study (LONZA KK, Cat No. PT-5006, Lot No. 18TL212639); T2DM-ADSCs, established by primary culture from adipose tissue extracted from a T2DM patient (39-year-old woman), was used in this study (LONZA KK, Cat No. PT-5008, Lot No. 1F4619).

### 4.3. Next-Generation Sequencing

The ADSC transplantation model used in this study was established in our preceding studies [29,30]. The cDNA domain (75 bp) and barcode sequence were analyzed by fragment analysis using the Illumina NextSeq.

### 4.4. The Isolation of Mitochondria

The transplanted tissues were homogenized in a buffer containing protease inhibitors. The homogenate was centrifuged at 1000× *g*, and the supernatant was collected. The supernatant was centrifuged at 12,000× *g* to collect a precipitate containing mitochondria. Mitochondria were purified by centrifugation at 1000× *g* using a 100 kDa size filter. The purity of the mitochondrial fraction is shown in the supplemental data (Supplemental Figure S1).

### 4.5. Mitochondrial DNA (mtDNA) Copies

The number of mtDNA copies was determined by four different genes (NADH dehydrogenase subunit 1 [ND1], solute carrier organic anion transporter family member 2B1 [SLCO2B1], ND5, and serpin family A member 1 [SERPINA1]) using the Human mtDNA Monitoring Primer Set. Genomic DNA was extracted by homogenizing transplants and using a NucleoSpin Tissue kit. The genomic DNA, four different primers in the Human mtDNA Monitoring Primer Set, and a PCR enzyme (SYBR Premix Ex Taq^TM^ II) were mixed for subsequent reaction. The reaction was performed using a quantitative PCR device (StepOne-Plus-01) under the following conditions: 95 °C for 30 s (Step 1), followed by 40 cycles of 95 °C for 5 s and 60 °C for 30 s (Step 2). The number of mtDNA copies was calculated as the mean of 2^ΔCt^ from the difference in the cycle threshold values of ND1/SLCO2B1 and ND5/SERPINA1.

### 4.6. Analysis of the Mitochondrial Membrane Potential

The mitochondrial membrane potential was analyzed using a JC-1 MitoMP Detection Kit. After treating transplants with trypsin and STEMxyme 2-collagenase, dissociated cells were primarily cultured and inoculated into an eight-well culture slide and incubated overnight under normal culture conditions in an incubator adjusted to 37 °C and 5% CO_2_. Following the addition of 100 µL of JC-1 working solution (4 µM), the sample was incubated for 30 min at 37 °C and 5% CO_2_. After washing the sample with HBSS, an imaging buffer solution was added. Finally, the sample was observed with a fluorescent microscope (green and red fluorescence at 488 nm and 561 nm wavelength, respectively).

### 4.7. Mitophagy Analysis

Mitophagy was analyzed using the Mtphagy Dye and Lyso Dye of the Mitophagy Detection Kit. After treating transplants with trypsin and STEMxyme 2-collagenase, primary culture cells were prepared. Cells were inoculated into a 96-well plate and incubated overnight under normal culture conditions in an incubator adjusted to 37 °C and 5% CO_2_. After washing the sample twice with a serum-free medium, the Mtphagy Dye working solution was added at 100 nM and incubated for 30 min at 37 °C and 5% CO_2_. To observe the colocalization of Mtphagy Dye and lysosome, 1 µM Lyso Dye working solution was added, and the sample was further incubated at 37 °C and 5% CO_2_. After washing the sample with Hanks’ HEPES buffer, staining of the mitophagy dye (Mtphagy Dye) and lysosome dye (Lyso Dye) were observed with a fluorescent microscope (green and red fluorescence at 488 nm and 561 nm wavelength, respectively). Following the confirmation of colocalization, the red fluorescence from the mitophagy dye (Mtphagy Dye) was measured at 561 nm wavelength using a fluorescence plate reader.

### 4.8. Nuclear Factor-kB (NF-kB) Reporter Assay

An NF-kB Secreted Alkaline Phosphatase Assay Kit was used to evaluate the NF-kB activity. Transplants were treated with trypsin and STEMxyme 2-collagenase to prepare primary culture cells. Cells were inoculated into a 96-well plate and incubated overnight under normal culture conditions in an incubator adjusted to 37 °C and 5% CO_2_. Cells were inoculated into a six-well culture plate at 5 × 10^5^ cells/well and incubated until they reached 80% confluence. A mixture of the pNF-kB/SEAP plasmid DNA (4 µg) and Lipofectamine LTX (10 µL) was added at 24-h intervals, and the cells were incubated for 72 h. Cells were collected and analyzed at 405 nm wavelength using the Secreted Alkaline Phosphatase (SEAP) assay to quantify the NF-kB reporter activity based on calibration curves.

### 4.9. Statistical Analysis

Results are presented as the mean ± standard deviation. Comparison between multiple groups was performed using two-way ANOVA with Tukey–Kramer post hoc tests, with *p*-values < 0.05, denoting statistical significance.

## Data Availability

The datasets generated during the current study are available in the Harvard Dataverse repository, https://doi.org/10.7910/DVN/DWOLNR.

## Supplementary Materials

The following supporting information can be downloaded at https://www.mdpi.com/article/10.3390/ijms26020719/s1.
