# Supplementary material for: The Diurnal Variation in Mitochondrial Gene in Human Type 2 Diabetic Mesenchymal Stem Cell Grafts
Source: Int J Mol Sci. 2025 Jan 16;26(2):719. doi: 10.3390/ijms26020719 (PMC11765740; doi:10.3390/ijms26020719)
Supplement: Supplementary file 1 [file ijms-26-00719-s001.zip › ijms-3349154-supplementary.pdf]

Supplemental Table S1

List of materials and instruments used in this study.

Reagent type	Designation (species)	Source	Location	Identifiers
Cell line	Adipose-Derived Stem Cells Normal (Homo sapiens)	Lonza	Basel, Switzerland	PT-5006
Cell line	Adipose-Derived Stem Cells T1DM (Homo sapiens)	Lonza	Basel, Switzerland	PT-5007
Medium	Adipose-Derived Stem Cells Growth Medium BulletKit™	Lonza	Basel, Switzerland	PT-4505
Reagent	0.05% Trypsin-EDTA (1x)	Gibco	Dublin, Ireland	25300-062
Reagent	Matrigel Basement Membrane Matrix For organoid formation	Corning	NY, USA	356255
Reagent	Matrigel Basement Membrane Matrix	Corning	NY, USA	354234
Machine	CO_2 incubator DIRECT HEAT INCUBATOR astec	ASTEC	Fukuoka, Japan	SCA-165D
mice	BALB / cAJcl-*nu / nu*	CLEA Japan	Tokyo, Japan	nu / nu
Kit	RNeasy Mini Kit (250)	QIAGEN Japan	Tokyo, Japan	74136
Reagent	NEBNext Poly(A) mRNA Magnetic Isolation Module	NEB	Tokyo, Japan	E7490
Kit	NEBNext Ultra II RNA Library Prep Kit for Illumina	NEB	Tokyo, Japan	E7770
Machine	Illumina NextSeq	Illumina	CA, USA	550
Software	CLC Genomics Workbench 12.0.3	QIAGEN Japan	Tokyo, Japan	12.0.3
Software	Database for Annotation, Visualization and Integrated Discovery	LHRI	MD, USA	12734009
Kit	PrimeScript™ RT reagent Kit	Takara Bio	Tokyo, Japan	RR037

	(Perfect Real Time)			A
Reagent	SYBR® Premix Ex Taq™ II (Tli RNaseH Plus)	Takara Bio	Tokyo, Japan	RR820A
Machine	StepOnePlus ™ Real-Time PCR System	Applied biosystems	CA, USA	4376592
Kit	Human mtDNA Monitoring Primer Set	Takara Bio	Tokyo, Japan	7246
Kit	NucleoSpin Tissue kit	MNA Takara Bio	Tokyo, Japan	U0952B
Kit	JC-1 MitoMP Detection Kit	Dojindo	kumamoto, Japan	MT09
Reagent	STEMxyme 2-Collagenase	Worthington	Lakewood, NJ	STZ2
Reagent	HBSS	Gibco	NY, USA	14175-079
Machine	fluorescent microscope BIOREVO	KEYENCE	Osaka, Japan	BZ-9000
Kit	Mitophagy Detection Kit	Dojindo	kumamoto, Japan	MD01
Kit	NF-kB Secreted Alkaline Phosphatase Assay Kit	Novus biologicals	CO, USA	NBP2-25286
Reagent	Lipofectamine™ LTX Reagent & Plus ™ Rreagent	Invitrogen	CA, USA	15338100
Machine	Infinite® 200 PRO	Tecan Japan	Kanagawa, Japan	200 PRO
Software	Prism9	GraphPad Inc.	CA, USA	academic ver.

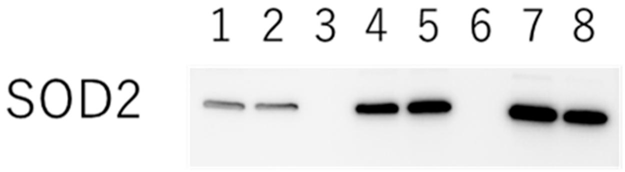

Supplemental Figure S1

Purity of mitochondrial fractions by Western blotting.

1. Whole Lysate N-ADSC (positive control)
2. Whole lysate T2DM-ADSC (positive control)
3. Non-mitochondrial fraction N-ADSC ZT10 (negative control)
4. Mitochondrial fraction N-ADSC ZT10
5. Mitochondrial fraction N-ADSC ZT22
6. Non-mitochondrial fraction T2DM-ADSC ZT10 (negative control)
7. Mitochondrial fraction T2DM-ADSC ZT10
8. Mitochondrial fraction T2DM-ADSC ZT22
